# Why is reusable bag consumption easier to say than do?

**DOI:** 10.3389/fpsyg.2022.956998

**Published:** 2022-09-23

**Authors:** Dongqing Yan, Xiang Cai, Meiying Xie, Sohail Ahmad Javeed, Fengqin Liu, Qun Cao

**Affiliations:** ^1^School of Business, Nanjing University of Information Science and Technology, Nanjing, China; ^2^School of Business, Guilin University of Electronic Technology, Guilin, China; ^3^School of Management, Hunan City University, YiYang, China; ^4^Sehan University, Yeongam-gun, South Korea; ^5^School of Business, Macau University of Science and Technology, Macau, China

**Keywords:** plastic ban, plastic consumption, reusable bag consumption, behavioral reasoning theory, attitude-behavior-context model, attitude-behavior gap

## Abstract

White pollution has become a global problem. China issued a strict plastic ban but fell into an awkward position. Despite the increasing environmental awareness, the positive attitude of consumers toward using reusable bags instead of plastic bags is difficult to reflect on from their behavior. This article bridges this gap by utilizing a consumer behavior framework based on the behavioral reasoning theory (BRT) and the attitude-behavior-context (ABC) model. This framework is tested using structural equation modeling with 481 Chinese consumers. This article confirms that the value has a significant impact on consumer attitudes. Meanwhile, the article reveals the positive influence of “reasons for” in predicting attitudes and the negative influence of “reasons against” in predicting intentions. Reusable bag consumption behavior is a result of multiple pathways working together, which causes the gap between attitudes and behaviors. This article also confirms the moderating role of the Chinese face and the enforcement of the plastic ban in influencing behavior. These findings offer interesting insights for enterprises and governments to solve the problem of plastic consumption.

## Introduction

The use of plastic products has caused a global crisis. Due to excessive use of plastic products, between 62 and 99 million metric tons of plastic waste are produced globally each year, threatening the environment in several ways (Lebreton and Andrady, 2019). Plastic waste takes a long time to decompose and fills up landfill sites quickly. In the environment, it also contributes to biodiversity loss through contamination of soil and water (Bharadwaj et al., 2021). According to the calculations of the Plastic Recycling Committee of the China Plastics Processing Industry Association, the Chinese use 1 billion plastic bags for grocery shopping every day, and more than 2 billion other plastic bags are used per day. In view of this, the State Council of the People's Republic of China issued the plastic restriction in 2008, which restricted the use of plastic shopping bags in production and sales. Since then, according to Table 1, from 2008 to 2020, several guidelines were issued to limit and ban plastic bags.

**Table 1:** 2008–2020 plastic restriction of China related to supporting policy sorting.

**Time**	**Issued by department**	**Name of policy**	**Primary coverage**
2008	The State Council of the People's Republic of China	Circular of The General Office of the State Council on Restricting the Production, Sale and Use of Plastic Shopping Bags	• Prohibit producing, selling, and using plastic bags with a thickness <0.025 mm • Pay for the use of plastic bags
2011	National Development and Reform Commission of the People's Republic of China, Ministry of Environmental Protection of the People's Republic of China	Notice on Launching a Special Campaign to Restrict the Production, Sale and Use of Plastic Shopping Bags	• Carry out special activities to restrict the production, sale, and use of plastic bags
2012	Ministry of Environmental Protection of the People's Republic of China	Regulations on Pollution Prevention and Control of Waste Plastics Processing and Utilization	• Make standards of processing and utilization of waste plastics
2013	National Development and Reform Commission of the People's Republic of China	Implementation of Deepening Restrictions on the Production and Sale of Plastic Shopping Bags	• Increase taxes on related businesses • Adjust raw materials
2014	National Development and Reform Commission of the People's Republic of China, Ministry of Finance of the People's Republic of China	Notice on Organizing and Implementing the Special Project of Biological Based Materials in 2014	• Organize the implementation of a special project on bio-based materials • Carry out a large-scale application of new products
2016	General Office of Environmental Protection of the People's Republic of China	Draft for Soliciting Opinions on Technical Requirements of Environmental Labeling Products	• Adjust production process • Adjust degradation properties • Adjust biological carbon content
2017	Ministry of Environmental Protection of the People's Republic of China, Development and Reform Commission of the People's Republic of China	Notice on Joint Cleaning and Consolidation of Recycling Industries such as Electronic Waste, Waste Tires, Waste Plastics, Waste Clothes and Waste Household Appliances	• Clean up and rectify recycling industries: electronic waste, waste tires, waste plastics, etc.
2018	Standing Committee of the National People's Congress	Environmental Protection Law of the People's Republic of China	• Adjust printing labels • Adjust storage of plastic
	Ministry of industry and Information Technology of the People's Republic of China	Industrial Specification conditions for Comprehensive Utilization of Waste Plastics	• Stipulate the standards of utilization and consumption of PET enterprises
2019	National Development and Reform Commission of the People's Republic of China	Index Catalog of Industrial Structure Adjustment	• Eliminate daily chemical products containing plastic microbeads.
2020	National Development and Reform Commission of the People's Republic of China, Ministry of Environmental Protection of the People's Republic of China	Opinions on Further Strengthening the Control of Plastic Pollution	• Reduce the consumption of disposable plastic products • Establish a management system for the production, consumption, and recycling of plastic

https://www.reportrc.com/article/20200509/6703.html

In the “Ten-Year Plastic Ban Research Report on Business Implementation” released by the NGO China Zero Waste Alliance in 2018, it was found that, among the 1,101 offline stores that conducted a survey of offline retail outlets in 9 places, including Beijing, Shenzhen, and Shenyang, 979 stores provided plastic bags. Meanwhile, there are only 89 stores that meet the requirements of full compliance, thickness standard, and charge, accounting for only 9.1% At the same time, only 36 companies, accounting for 3.7%, were able to comply with all the provisions of the ban. The implementation of the plastic ban is stuck in a quagmire; the plastic ban has existed in name only.

Therefore, the National Development and Reform Commission of the People's Republic of China and the Ministry of Environmental Protection of the People's Republic of China jointly issued the Opinions on Further Strengthening Plastic Pollution Control on 19 January 2020, which clearly proposed to strengthen the treatment of plastic pollution. The Opinions specify a timetable and roadmap for the replacement and banning of plastic products and strive to establish a whole-process supervision system of plastic products by 2025 so that plastic pollution can be effectively controlled. The guideline is seen as an upgraded version of the 2008 regulation on plastic bags, resulting in the strictest plastic ban in history.

The Policy Research Center for Environment and Economy of the Ministry of Ecology and Environment of the People's Republic of China publicly released the Citizens' Ecological and Environmental Behaviour Survey Report (2020)^1^ to the public, pointing out that the Chinese citizens believe that their own environmental behavior is important to protect the ecological environment, and their awareness of environmental protection has been greatly improved compared with previous years, but the proportion of consumers who choose to bring their own shopping bags or use reusable bags when shopping was found to be extremely low in the survey for shopping consumption. In the context of the strict implementation of the plastic ban and the increasing awareness of environmental protection among citizens, consumers prefer plastic bags rather than reusable bags for consumption. This phenomenon of inconsistency between words and actions deserves deeper investigation. The *status quo* of reusable bag consumption, easy to know and difficult to implement, is not properly addressed and will hinder the process of sustainable development.

The marginal contributions of this article are as follows. First, previous studies (Alzubaidi et al., 2021; Huang and Qian, 2021) mainly focused on the relationship between green consumption attitude and green consumption behavior under the traditional behavioral theory. Although some scholars (Park and Lin, 2020; Dhir et al., 2021) discussed the gap between green consumption attitude and behavior, there are few studies on the gap between reusable bag consumption attitude and behavior in China. This study, for the first time, takes China's plastic ban as the background to further explore the reasons for the discrepancy between words and deeds in reusable bag consumption. Second, the traditional theory of rational behavior is no longer suitable for the current issue; China is a country of strong social ties, and behavior does depend on not only attitudes but also the context in which consumption occurs (Shi et al., 2012). Therefore, the present study innovatively integrates the attitude-behavior-context (ABC) model and behavioral reasoning theory (BRT) into one theoretical model to construct the mechanism model of influencing reusable bag consumption and explores the mechanism of individual reusable consumption. Third, this study reveals the positive influence of “reasons for” in predicting attitudes and the negative influence of “reasons against” in predicting intentions. Reusable bag consumption behavior is a result of multiple pathways working together, which causes the gap between attitudes and behavior. This study also confirms the moderating role of the Chinese face and the enforcement of the plastic ban in influencing behavior. These findings offer interesting insights for enterprises and governments to solve the problem of plastic consumption.

The present study is structured as follows. The “Theories and research framework” section details the theoretical foundations and research framework regarding reusable bag consumption. In the “Research hypothesis” section, different hypotheses about reusable bag consumption are developed and discussed. The “Research method” section presents the design of the research scale and the basic information of the questionnaire in this article. The “Data analysis” section introduces the concrete steps of the structural equation model in detail. The “Discussion” section provides a discussion based on empirical studies. The “Conclusion” section summarizes the different theoretical and practical implications and the limitations and directions for future research.

## Theories and research framework

### Theory of planned behavior

There are several theories on whether consumers adopt or accept new products or services, such as the diffusion of innovation theory (DOT), the theory of reasoned action (TRA), and the theory of planned behavior (TPB) (Huang and Qian, 2021). The value-belief-norm (VBN) theory of Stern and TPB of Ajzen are widely used in green consumption (Kaiser et al., 2005). VBN emphasizes the role of values on individual behavior norms. This theory highlights the internal subjective performance of individual behavior. However, consumers face many external constraints when they make purchase decisions. Therefore, VBN ignores the influence of the objective environment on individual behavior (Wang and Du, 2016). More scholars have chosen TPB for consumer behavior research, which advocates that attitude, subjective norms, and perceptual control jointly influence behavioral intention and thus determine human behavior (Alzubaidi et al., 2021). These scholars believe that TPB extends VBN by incorporating objective constraints into subjective performance. For example, when using TPB to explore green consumption, it is argued that both subjective norms and perceived behavioral control influence consumers' intention to purchase green products (Liu et al., 2020).

### Behavioral reasoning theory

The behavioral reasoning theory emphasizes the connection function of rationality among individual values, attitudes, behavioral intentions, and behaviors (Westaby et al., 2010), which is the reason for individuals to reject or perform behaviors. According to this theory, rationality can not only affect individual intention through attitude but also directly affect individual behavior (Gifford and Chen, 2017; Dhir et al., 2021). Compared with other theories mentioned earlier, BRT not only focuses on the relationship between individual performance reason, attitude, and behavior but also highlights the influence of reasons against attitude and behavior, enriching theoretical research on the relationship between individual attitude and behavior (Sahu et al., 2020). At this stage, BRT has not been applied much, and there are few studies on green consumption, which mainly focus on energy, organic food, e-waste recycling, and other fields related to green products (Tandon et al., 2020; Dhir et al., 2021), and especially few studies on reusable bag consumption.

According to BRT, consumer attitude toward reusable bag consumption is the main predictor of consumer behavior intention (Kaur et al., 2020; Sahu et al., 2020). Rationality is the key factor in BRT (Westaby et al., 2010). If individuals harbor strong reasons for or against green consumption, they will have different influences on behaviors (Westaby, 2005).

According to the contractual level theory people have a cool attitude towards things at a great psychological distance, which is the main, the core, essence, and characteristics of the background to represent things, and to things about psychological distance tend to use low contractual level characterization, and play a secondary, auxiliary, marginalization, details, and background features to represent things (Fujita et al., 2008). When using the consumers' behavior far distance reusable bags model, consumers tend to be objective and calm and tend to produce the performance of plastic consumption. When the distance is close, consumers tend to be subjective and are more likely to have reasons for rejection (Griskevicius et al., 2010). This theory further verifies the necessity and importance of the rationality of BRT.

### Attitude-behavior-context model

The attitude-behavior-context model suggests that the relationship between attitude (A) and behavior (B) depends on context (C) (Guagnano et al., 1995). When the contextual factors are strongly positive or negative, they effectively promote or inhibit the occurrence of behaviors, and the correlation between attitudes and behaviors is weak (Stern, 2000; Xu et al., 2017). When a certain behavior is difficult to realize and requires a high cost in terms of time, experience, or money, then the individual's attitude will not necessarily lead to the realization of the behavior (Olander and Thogersen, 2005). Therefore, behavior is the output of the interaction between individual attitudes and contextual factors (Guagnano et al., 1995). All the time, the indicators of situational factors include social culture, group expectations, advertising, government regulations, and other legal and institutional factors (Stern, 2000). ABC is most commonly used in the context of a family or a social organization. For example, Xu et al. (2017) chose group norms and organizational support as situational variables to study the energy-saving behavior of office employees and found that the interaction between attitude and situational variables has an important impact on behavior.

The attitude-behavior-context model assumes that behavior is the culmination of the interaction between individual attitudes and contextual factors (Guagnano et al., 1995). Consumer behavior is not only affected by consumer attitudes but also by external contextual factors (Wang et al., 2018). Contextual factors refer to the special environment that consumers are exposed to in their consumption behaviors, including both material factors and social factors (Stern, 2000). Specifically, contextual factors include interpersonal relationships, group expectations, advertising, government regulations, and other legal and institutional factors (Xu et al., 2017).

### Research framework

Although TPB suggests that there is a strong correlation between attitudes and behavioral intentions (Chen and Tung, 2014; Liu et al., 2020), according to the Citizens' Ecological Environmental Behaviour 2020 survey report, it was found that there is a gap between attitudes and behaviors, that is, strong attitudes but presenting weak behaviors or weak attitudes but presenting strong behaviors (Park and Lin, 2020), which traditional theory does not explain. BRT organically integrates individuals' values, behavioral rationality, attitudes, and intentions. In addition, it can integrate the reasons for or against reusable bag consumption into one framework, which is conducive to understand the reasons for the formation of the attitude-behavior gap.

Furthermore, the influence of contextual factors cannot be ignored because of the pro-environment behavior of plastic consumption. It is important to emphasize that China has high cultural semantics and strong social relationships; thus, contextual variables have a greater impact on the consumption behavior than in Western countries. Therefore, only an in-depth exploration of the role of contextual variables and a study of consumers as real, complex people in certain contexts can enhance the consistency of surrogate shaping of consumer attitudes and behaviors. ABC can further explain the manifestations and reasons for the gap.

**Figure 1:**
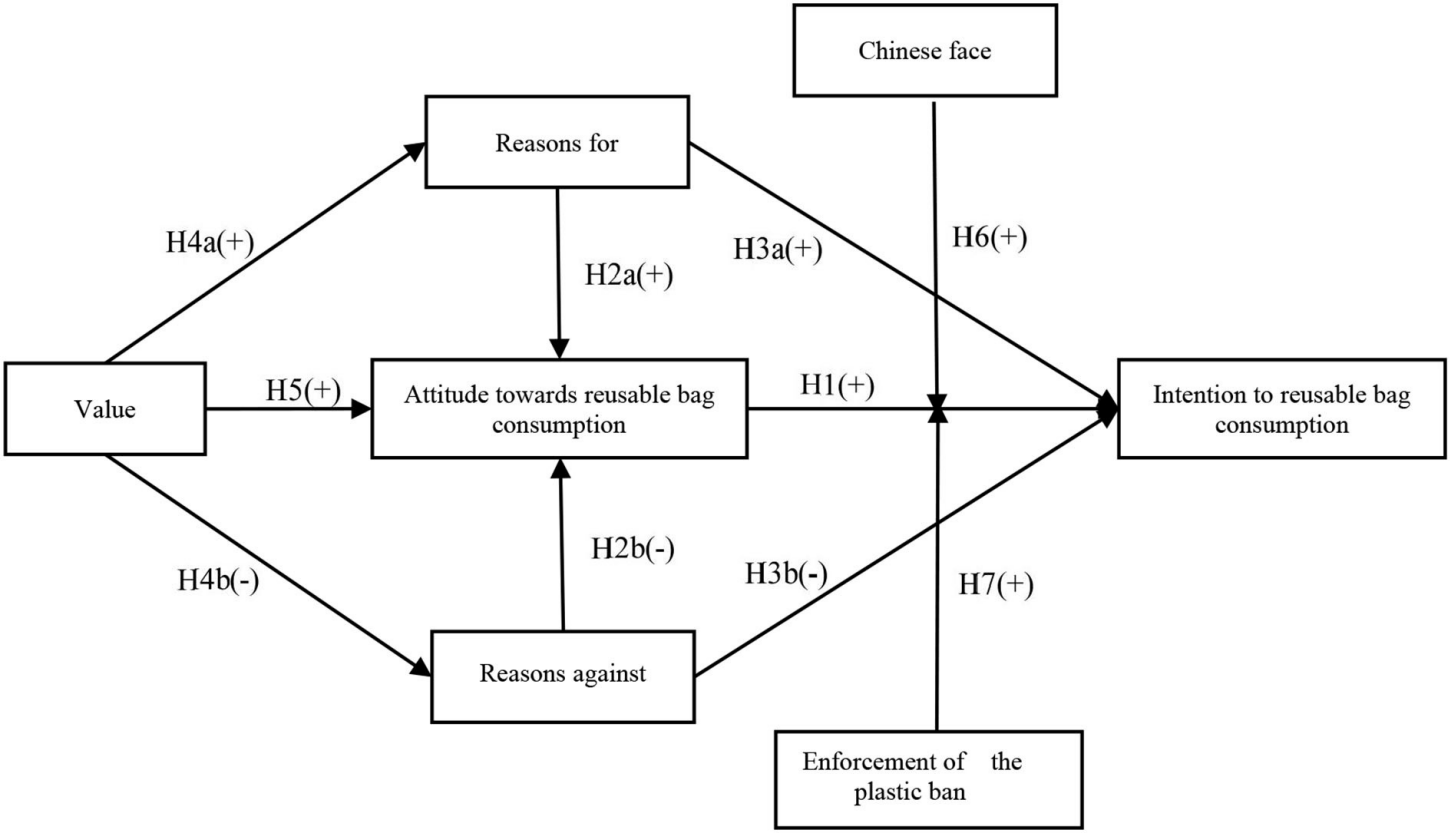
Research framework diagram.

Based on the earlier discussion, this study proposes the research framework, as seen in Figure 1.

## Research hypothesis

### Attitudes and intentions

It is proposed that attitude is a key and effective predictor of behavioral intentions (Ajzen, 1991; Westaby et al., 2010). Due to its high accuracy, behavioral intention prediction is widely used in consumer behavior research. Behavioral intention as the most effective predictor of human behavior has been widely certified by the academic circle. (Dhir et al., 2021). Therefore, this study takes the intentions of reusable bag consumption as a proxy variable of behaviors. The nature of reusable bag consumption is a kind of green consumption. While the correlation between the attitude and behavior of green consumption has been supported by various sources, including the energy field (Litvine and Wüstenhagen, 2011), green food (Gifford and Chen, 2017), and e-waste recycling (Dhir et al., 2021), there are few studies on plastic reusable bag consumption. At the same time, as previously mentioned, in the context of China having formed the strict “plastic restriction,” the Citizens' Ecological Environmental Behaviour Survey Report (2020) (see text footnote 1) pointed out that the Chinese citizens consider their own environmental behavior important to protect the ecological environment, and their environmental awareness has greatly increased compared to previous years; meanwhile, their attitudes toward plastic reusable bag consumption have also greatly improved. Therefore, this study proposes that consumers' positive attitudes toward plastic reusable bag consumption may promote their behavior. Accordingly, we propose the following hypothesis:

**H1** Attitudes toward reusable bag consumption share a positive association with reusable bag consumption intentions.

### Rationality, attitude, and intentions

Compared with other theories, BRT emphasizes the effective role of rationality in predicting behaviors (Dhir et al., 2021). For a particular behavior, researchers observed that rationality can act as either a facilitator or a disincentive, influencing consumer cognition (Claudy and O'driscoll, 2013). Regarding reusable bag consumption, the rationality of contextualization, which includes reasons for performing reusable bag consumption and reasons against reusable bag consumption, is an important predictor of attitude and behavioral intentions.

The existing literature on consumer behavior using BRT mainly incorporates personal and environmental interests into the dimension of reasons for fulfilling consumer behavior (Dwivedy and Mittal, 2013; Botelho et al., 2016). Personal interests refer to the economic or non-economic benefits gained by participating in the consumption of reusable bags (Pillai and Sivathanu, 2018). Previous studies focused on economic benefits, but since the rationality of plastic replacement behavior is not limited to the reduction of economic expenses but also includes personal health (Dhir et al., 2021), the present study focuses on non-economic benefits in rationality. Environmental benefits included energy savings, reduced white pollution, and improved air quality. Reusable bag consumption can reduce the use of plastic products and environmental pollution. Consumers consider the hazards of plastic products when consuming them and choose shopping bags that can replace plastic products, such as eco-friendly bags, which bring great environmental benefits (Bharadwaj et al., 2021). Several scholars showed that fulfillment reasons are positively related to consumer attitudes (Claudy et al., 2015). Some scholars (Botelho et al., 2016) confirmed a positive contribution of personal benefits to consumer attitudes about e-waste recycling and management, and other scholars (Bharadwaj et al., 2021) illustrated the positive effect of environmental benefits on attitudes. This study argues that the personal and environmental benefits from the implementation of reusable bag consumption will enable consumers to understand the positive role of reusable bag consumption and have a positive attitude. Accordingly, we propose the following hypothesis:

**H2a** “Reasons for” share a positive association with the attitude toward reusable bag consumption.

“Reasons against” refer to an individual's negative reaction to a specific behavior (Sahu et al., 2020). Previous studies assumed that “reasons against” consumption include five types, namely, use, value, risk, image, and traditional cognition (Claudy et al., 2015; Gupta and Arora, 2017). Considering the nature of reusable bag consumption as a green consumption activity, the study includes risk barriers, use barriers, and value barriers in the “reasons against.” Risk barrier refers to the risk of food leakage, while the easy breakage of reusable bags is a separate concern. The value barrier refers to the difference between the money that consumers think they get and the money they actually get (Talwar et al., 2020). In the study, the use barrier is the contradiction between the emerging reusable bag consumption and the traditional plastic use behavior of consumers, leading to their lack of positive adoption of reusable bag consumption. Previous studies showed that “reasons against” are negatively correlated with consumer attitudes and intentions (Claudy et al., 2015; Gupta and Arora, 2017). Risk perception has a significant impact on consumer behavior, showing a negative correlation (Kaur et al., 2020; Dhir et al., 2021). Some scholars (Kaur et al., 2020) confirmed a negative association between barriers to use and payment intention in mobile payments. Talwar et al. (2020) also found that there was a significant negative correlation between the value barrier and purchase intention of online travel agencies. Similarly, some scholars (Kushwah et al., 2019) highlighted the negative impact of value barriers on consumers' intention to consume organic food. Therefore, there may also be reasons against reusable bag consumption in the negative impact on consumer attitudes. Accordingly, we propose the following hypothesis:

**H2b** “Reasons against” share a negative association with intentions toward reusable bag consumption.

It was the first to propose that consumers seek mental shortcuts rather than affecting their behavior through attitudes (Tversky and Kahneman, 1974). In addition, scholars based on BRT found that rationality bypasses the mediating role of attitudes and directly influences behavioral intentions (Westaby, 2005). While attitudes represent personal likes and dislikes, rationality is the reason for adopting a particular product or not. It is common for consumers to directly influence consumption behavior by having the reason for adopting reusable bag consumption. For example, although consumers have a positive attitude toward reusable bags, the high price is the main reason why most consumers refuse to purchase reusable bags. In addition, some scholars (Claudy et al., 2015) confirmed the direct influence of rationality on behavior in a study on car sharing, while some scholars (Talwar et al., 2020; Dhir et al., 2021) highlighted the direct influence of “reasons against” in rationality on consumer behavior. Accordingly, we propose the following hypotheses:

**H3a** “Reasons for” share a positive association with intentions toward reusable bag consumption.
**H3b** “Reasons against” share a negative association with intentions toward reusable bag consumption.

### Values, rationality, and attitude

Values are rooted in life and reflect the subjective initiative of individuals toward the objective world (Schwartz, 1994). This abstract motivation structure will promote the completion and realization of goals. Values guide the selection of actions and the evaluation of people and things through the connection with these abstract goals. As a component of the value system, the value of reusable bag consumption originated from the VBN (Stern, 2000). Therefore, there is a correlation between the value and rationality of reusable bag consumption (Claudy et al., 2015; Tandon et al., 2020). For example, at this stage, the state has formulated such a strict plastic restriction and joint multi-sectoral publicity, the fundamental purpose of which is to make the public establish the correct values of plastic consumption. We, therefore, believe that there is a positive association between values and reasons for reusable bag consumption and a negative association between values and reasons against reusable bag consumption.

In addition, VBN, from the perspective of the relationship between humans and nature, holds that individuals tend to express their environmental protection claims through purchase and consumption behaviors (Haws et al., 2014). As a result, consumers who value reusable bag consumption tend to have positive purchase attitudes toward environmental attributes (Nguyen et al., 2016). Accordingly, we propose the following hypotheses:

**H4a** Value shares a positive association with “reasons for” toward reusable bag consumption.
**H4b** Value shares a negative association with “reasons against” toward reusable bag consumption.
**H5** Value shares a positive association with the attitude toward reusable bag consumption.

### Chinese face and the enforcement of the plastic ban

Studies found that all Buddhism, Taoism, and Confucianism cultures, the culture of “unity of nature and man,” and the group culture in the Chinese social and cultural context have a positive impact on consumer attitudes and behaviors toward green products (Wang et al., 2018). This is expressed in terms of one's image in the eyes of others and the morality of one's own behavior, as well as the social effects it produces, such as “face” (Juan Li and Su, 2007), and can be obtained through interpersonal interactions that are contextual in nature (Ho, 1976; Qi, 2011). For example, there is often a group effect, whereby others in the group choose to consume vicariously, and then, the likelihood of individuals choosing to consume vicariously is greatly increased. Since ancient times, the face has been the spiritual program of the Chinese, and the culture of the face is widespread in China, which is deeply involved in every aspect of the Chinese social life. We, therefore, treated the Chinese facial culture as an important element of the social context and a moderating variable. Some scholars, in their studies on face perception, both found that the stronger the Chinese face, the stronger the intention of people to engage in reusable bag consumption behavior.

Attitude-behavior-context holds that contextual factors include interpersonal influences, group expectations, advertising, government regulations, and other legal and institutional factors (Stern, 2000). As a legal system, the promulgation of the plastic restriction is an important step toward attaching the importance to environmental protection consumption in China, and the promotion effect of the plastic restriction on consumers' behavior of reusable bag consumption has also been confirmed (Gupta and Arora, 2017; Bharadwaj et al., 2021). Therefore, we treated the enforcement of plastic restriction as one of the moderating variables. Accordingly, we propose the following hypotheses:

**H6** The Chinese face positively moderates the associations between attitude with intentions to engage in reusable bag consumption.
**H7** The enforcement of the plastic restriction positively moderates the associations between attitudes and intentions to engage in reusable bag consumption.

## Research method

### Questionnaire design

The measurement scales for reusable bag consumption draw on those that have been used in previous studies. The measurement of the values of reusable bag consumption refers to the scale used in studies by scholars (Haws et al., 2014). The rationality of behavior was divided into reasons for reusable bag consumption (economic benefits and environmental benefits) and reasons against reusable bag consumption (risk barriers, use barriers, and value barriers). The measurement of “reasons against” draws on the scale used by some scholars (Kaur et al., 2020; Tandon et al., 2020), and the consumption of “reasons for” refers to the scale used in the study by scholars (Claudy et al., 2015). The measurement of attitudes and intentions toward reusable bag consumption draws on the scales used in the study by some scholars (Wang et al., 2016). The Chinese face is based on the scale used in some studies (Bao et al., 2003). In addition, the scale of the enforcement of the plastic ban, as a public policy, is based on the evaluation system proposed by scholars. This questionnaire is a five-point Likert scale, ranging from 1 (completely agree/knowledge) to 5 (completely disagree/do not know). In this study, the abovementioned scale is appropriately modified according to the needs of the study, as seen in Table 2.

**Table 2:** Measurement scale related to generation plastic consumption (Lebreton and Andrady, 2019).

**Latent variables (Reference)**	**Question items**
Consumer values (Haws et al., 2014)	VAL1: When I consume, it's important for me to use reusable bags
	VAL2: When I consume, I consider the potential impact of my behavior on the environment
	VAL3: When I consume, My shopping habits are influenced by my environmental awareness
	VAL4:When I consume, I worry about the waste of resources caused by using plastic
	VAL5: When I consume, I consider myself environmentally responsible
	VAL6: When I consume, I am willing to pay extra for environment-friendly behavior by using the reusable bag
Reason against (Kaur et al., 2020; Tandon et al., 2020)	RA1: I choose plastic bags every time because the unit price of plastic bags is low
	RA2: I choose plastic bags every time because they are easy to carry and get
	RA3: I choose plastic bags for the reason that they give me a better experience each consumption
Reason for (Claudy et al., 2015)	RF1: I refuse plastic bags because they can be used many times and the average cost is low
	RF2: I refuse plastic bags every time I buy them because plastic is bad for my health
	RF3: I refuse plastic bags every time I consume them in order to reduce environmental pollution
Attitude toward reusable bag consumption (Wang et al., 2016)	ATT1: I think using reusable bags is a good experience
	ATT2: I think it's good to use reusable bags
	ATT3: I think we should try our best to promote the use of reusable bags
Chinese face (Bao et al., 2003)	CF1: I'm worried that using plastic bags will make me look environmentally insensitive
	CF2: I'm worried that using reusable bags will make me look economical and mean
	CF3: I feel guilty about not using reusable bags if everyone around me uses them
Enforcement of the plastic ban (Fan and Zhan, 2014)	EP1: I know the implementation of the plastic restriction
	EP2: I understand the details of the ban
	EP3: I think it is difficult to enforce the plastic restriction
	EP4: I think the national plastic restriction policy is strictly implemented
Intention to reusable bag consumption (Wang et al., 2016)	INT1: I would like to learn about why the use of plastic products is restricted
	INT2: I would like to recommend my friends and relatives to use reusable bags
	INT3: I'm willing to use reusable bags when I consume

### Questionnaire survey and collection

Before the final version of this questionnaire, 30 consumers were evaluated using an online pilot study. These 30 consumers are from a group purchasing from China Resources Supermarket. Since the members of the group are responsible for the daily expenses of the family, they need to purchase a lot of goods and they have reusable bag consumption, so they are suitable research objects. Participants were asked to read and evaluate the survey questions, which were formally distributed after it was determined that they did not have any unclear, confusing, or meaningless items to ask. This study adopted an online research-based approach, supplemented by offline research. The online research mainly sent questionnaires through a special questionnaire design platform and invited consumers from different groups purchasing from supermarkets to fill out the questionnaires through QQ, WeChat, and e-mail communication software, and a total of 503 questionnaires were collected. An offline survey was conducted in Nanjing Pukou Market on 15 May 2021, Nanjing Rsun Square on 19 May 2021, and Nanjing Xin Jiekou on 2 June 2021. Notably, 97 questionnaires were collected. A total of 600 questionnaires were collected in this research, in which 119 invalid ones were excluded because these questionnaires were filled in a too short time, inconsistent, and repeated, and 481 valid ones remained, with a recovery rate of 80.1% (refer to Table 3 for the basic information). As can be seen from the table, the respondents of this research are concentrated in those who are 20–50 years old, have a higher education, and have a certain economic ability. Thus, this shows that these respondents have a certain understanding of the relevant information about the country's formulation of a series of strict plastic regulations.

**Table 3:** Demographic profile of the participants.

**Variable**	**Category**	**Frequency**	**Percentage**
Gender	Male	223	0.46
	Female	258	0.54
Age	Under 20	33	0.07
	21–30	216	0.45
	31–40	126	0.26
	41–50	79	0.16
	50 or older	27	0.06
Academic qualifications	High school or technical secondary school (including) below	56	0.11
	College	102	0.21
	Undergraduate course	166	0.35
	Master degree or above	157	0.33
Income(RMB)	3,000 of the following	120	0.25
	3,000–5,000	82	0.17
	5,000–8,000	112	0.23
	8,000–10,000	94	0.19
	More than 10,000	73	0.15
Job	Education	149	0.31
	Services	29	0.06
	Freelancer	24	0.05
	Company employees	101	0.21
	Government staff	38	0.08
	Students	140	0.29
	East	240	0.50
Region	Middle	145	0.30
	West	96	0.20

## Data analysis

### Common method bias

Common method bias refers to artificial co-variation between predictor and effector variables due to the same data source or rather, the same measurement environment, the item context, and the characteristics of the item itself (Zhao et al., 2010). To mitigate potential bias from the above sources of bias, we took different steps to ensure that the common method bias would not significantly affect the study design and results. First, the respondents were clearly informed when the questionnaire was issued that the survey was conducted anonymously and there was no standard answer, and they were emphasized to fill in truthfully and reduce the probability of deviation through procedural control (Podsakoff et al., 2003; Dhir et al., 2021). Second, Harman's single factor test (Harman, 1976) is often used to test whether the common method bias is serious (Podsakoff et al., 2003). After testing the data of this survey in Table 4, it was found that the proportion of the first common factor was <40%, and there was no serious common method bias.

**Table 4:** Common method bias test.

**Total variance interpretation**					
**composition**	**Initial eigenvalue**			**Extract the sum of squares of loads**		
	**A total of**	**Percentage of variance**	**Cumulative %**	**A total of**	**Percentage of variance**	**Cumulative %**
1	8.864	35.456	35.456	8.864	35.456	35.456
2	4.198	16.793	52.249	4.198	16.793	52.249
3	1.969	7.875	60.123	1.969	7.875	60.123
4	1.525	6.101	66.225	1.525	6.101	66.225
5	1.191	4.762	70.987	1.191	4.762	70.987

### Confirmatory factor analysis

In this study, the performance of the measurement model was evaluated by validation factor analysis. Table 5 shows the consistency and validity among the question items and dimensions. The unstandardized factor loadings of the question items were all >0.6, and the results of the confirmatory factor analysis showed that all factor loadings were significant at a *p*-value = 0.01. Meanwhile, the composite reliability and Cronbach's alpha exceeded the critical value of 0.7, indicating good reliability of the scale (Hayes, 2009). In addition, the average variance extracted exceeded the critical value of 0.5, indicating that the scale had good convergence validity. Table 6 further shows that the diagonal value is the square root value of the average variance extracted, and the root is greater than the correlation coefficient between potential variables, indicating that the internal correlation between observed variables is greater than the external correlation (Fornell and Larcker, 1981), demonstrating that the scale has good discriminant validity.

**Table 5:** Reliability test.

**Item**	**Parameters of significant**				**Item Reliability**	**Composite Reliability**		**Convergence validity**
	**Est**	**S.E**	**Est/S.E**	**P**	**R-square**	**CR**	**Cronbach's alpha**	**AVE**
VAL1	0.727	0.023	31.068	<0.01	0.529	0.903	0.879	0.631
VAL2	0.837	0.016	52.309	<0.01	0.701			
VAL3	0.855	0.015	57.903	<0.01	0.731			
VAL4	0.799	0.019	42.651	<0.01	0.638			
VAL5	0.821	0.017	47.702	<0.01	0.674			
VAL6	0.717	0.024	29.548	<0.01	0.514			
RA1	0.907	0.011	83.363	<0.01	0.823	0.933	0.797	0.823
RA2	0.899	0.012	77.702	<0.01	0.808			
RA3	0.916	0.01	88.111	<0.01	0.839			
RF1	0.843	0.016	52.742	<0.01	0.711	0.853	0.802	0.767
RF2	0.882	0.014	64.945	<0.01	0.778			
RF3	0.901	0.012	72.106	<0.01	0.811			
ATT1	0.775	0.021	36.258	<0.01	0.601	0.89	0.727	0.73
ATT2	0.874	0.014	60.55	<0.01	0.764			
ATT3	0.909	0.013	70.636	<0.01	0.826			
INT1	0.846	0.016	52.211	<0.01	0.716	0.912	0.803	0.775
INT2	0.921	0.012	77.286	<0.01	0.848			
INT3	0.873	0.014	61.546	<0.01	0.762			
PO1	0.918	0.01	90.555	<0.01	0.843	0.917	0.822	0.734
PO2	0.926	0.01	95.904	<0.01	0.857			
PO3	0.77	0.02	37.851	<0.01	0.593			
PO4	0.803	0.018	44.291	<0.01	0.645			
CF1	0.771	0.024	32.487	<0.01	0.594	0.834	0.846	0.627
CF2	0.821	0.021	38.746	<0.01	0.674			
CF3	0.782	0.023	33.835	<0.01	0.612			

Estimator, Est; S.E, Standardized Estimator; Composite reliability, CR; Average variance extracted, AVE; Attitude, ATT; Reasons for, RF; Reasons against, RA; Intentions, INT; Value, VAL; Chinese face, CF; Enforcement of the plastic ban, EP.

**Table 6:** Validity test.

**Dim**	**Discriminate validity**						
	**VAL**	**RA**	**RF**	**ATT**	**CF**	**EP**	**INT**
VAL	**0.791**						
RA	0.454	**0.907**					
RF	0.743	0.389	**0.880**				
ATT	0.675	0.343	0.647	**0.854**			
CF	0.637	0.537	0.572	0.632	**0.88**		
EP	0.218	0.008	0.110	0.170	0.218	**0.857**	
INT	0.228	0.027	0.097	0.063	0.110	0.559	**0.792**

The bold values on the diagonal are square root values of AVE, and the lower triangle is the Pearson correlation of the dimensions.

### Model fit test

The fitting test is used to test the prediction model and verify the accuracy of its prediction results, mainly through the fitting index values in the table (Barrett, 2007). This structural model also returned a good model fit: Chi square (CMIN) = 360, degree of freedom (DF) = 126, comparative fit index (CFI) = 0.97, tucker-Lewis index (TLI) = 0.96, root-mean-square error of approximation (RMSEA) = 0.067, standardized root mean square residual (SRMR) = 0.058. The specific test results of the model are shown in Table 7, which all meet the requirements of the index values.

**Table 7:** Model fit test.

**Fit index**	**Key value (recommended value)**	**Model indexes**	**Conform to the (Support)**
MLX^2^	the smaller the better	360	
DF	The bigger the better	126	
*X*^2^/*DF*	*1* < *X*^2^/*DF* < *3*	2.8	Yes
CFI	>0.9	0.97	Yes
TLI	>0.9	0.96	Yes
RMSEA	<0.08	0.067	Yes
SRMR	<0.08	0.058	Yes

### Hypothesis test

The above hypotheses were tested by structural equation modeling, and the results of the study largely supported the model proposed in this study. Specifically, as seen in Table 8, among these ten hypotheses, eight hypotheses were confirmed (H1, H2a, H3b, H4a, H4b, and H5) and two hypotheses were not confirmed (H2b and H3a).

**Table 8:** Hypothesis testing.

**DV**	**Std.Est**.	**S.E**.	**Est/S.E**.	***P*-value**	**Hypo (Support)**
H1	0.329	0.058	5.634	<0.01	Yes
H2a	0.319	0.063	5.106	<0.01	Yes
H2b	0.036	0.043	0.84	0.401	No
H3a	0.082	0.068	1.2	0.23	No
H3b	0.29	0.044	6.632	<0.01	Yes
H4a	0.747	0.025	30.186	<0.01	Yes
H4b	0.452	0.04	11.355	<0.01	Yes
H5	0.416	0.064	6.463	<0.01	Yes
H6	0.208	0.403	4.801	<0.01	Yes
H7	0.155	0.037	4.191	<0.01	Yes

### Mediation analysis

To further test the mediation effect of the model, the present study follows the procedure of mediation effect analysis proposed by some scholars (Zhao et al., 2010) and conducts the bootstrap mediation variable test with a sample size of 5,000 and a 95% confidence interval by referring to the multiple parallel mediation variable testing methods proposed by some scholars (Preacher and Hayes, 2008). The results are seen in Table 9. Among them, three paths, namely, values → reasons for reusable bag consumption → intentions, consumption values → consumption attitude → intentions, and values → reasons against reusable bag consumption → attitudes → intention have mediation effects because LLCI—ULCI does not contain 0. In contrast, two paths, namely, LLCI–ULCI of values → reasons for reusable bag consumption → attitude → intentions and values → reasons against reusable bag consumption → intentions, contain 0, and the mediation effect does not exist.

**Table 9:** Mediation test.

**Path**	**Point Estimate**	**Product of Coefficients**			**Bootstrap 5,000 times 95% CI**	
					**Bias**	**corrected**
		**S.E**.	**Est/S.E**.	***P*-value**	**Lower**	**Upper**
Indirect effects						
VAL → RA → INT	0.108	0.024	4.484	0.000	0.074	0.183
VAL → RA → ATT → INT	0.003	0.006	0.496	0.620	−0.008	0.020
VAL → ATT → INT	0.114	0.048	2.386	0.017	0.051	0.287
VAL → RF → ATT → INT	0.064	0.032	1.980	0.048	0.024	0.182
VAL → RF → INT	0.049	0.054	0.897	0.370	−0.038	0.199

### Moderated mediation analysis

The bootstrap method was adopted to test the moderated mediation model (Hayes, 2009; Wang et al., 2020), and the results are shown in Table 10. Through the sample test, both the Chinese face and the enforcement of the plastic ban can positively moderate the relationship between the attitude toward reusable bag consumption and the intention through the mediation effect.

**Table 10:** Moderated mediation model.

**path**	**Moderator**	**Product of Coefficients**			**95% CI**
					**Bias**	**corrected**
		**EST**	**S.E**.	***P*-value**	**Lower**	**Upper**
VAL → ATT → INT	CF					
	Low level (−1SD)	0.026	0.029	0.366	0.007	0.066
	High level (+1SD)	0.256	0.102	0.012	0.183	0.43
	EP					
	Low level (−1SD)	0.059	0.063	0.349	0.023	0.175
	High level (+1SD)	0.223	0.038	0.000	0.220	0.256
VAL → RF → ATT → INT	CF					
	Low level (−1SD)	0.015	0.023	0.496	0.008	0.049
	High level (+1SD)	0.150	0.029	0.000	0.119	0.175
	EP					
	Low level (−1SD)	0.035	0.017	0.039	0.023	0.06
	High level (+1SD)	0.131	0.045	0.003	0.071	0.181

In the pathway of values → attitude → intentions, the bias-corrected interval under low Chinese face (−1SD) includes 0, so the mediation effect of this pathway under low face culture is not significant, while the bias-corrected interval under high Chinese face (+1SD) does not include 0, and the mediation effect is significant. Thus, the Chinese face has a moderation effect on the mediation effect of this path, as seen in Table 10. Meanwhile, the bias-corrected interval under both low government enforcement (−1SD) and high government enforcement (+1SD) does not include 0, so the mediation effect is significant. However, it can be found in the table that the effect value under high government enforcement (0.223) is significantly higher than the effect value under low government enforcement (0.059), as seen in Table 10, so the present study concludes that the moderation effect of plastic restriction enforcement on the mediation effect of this path exists.

In the pathway of values → reasons for reusable bag consumption → attitude → intentions, the bias-corrected interval under low Chinese face (−1SD) includes 0, so the mediation effect of this pathway under low face culture is not significant, while the bias-corrected interval under high Chinese face (+1SD) does not include 0 and the mediation effect is significant. Thus, the Chinese face has a moderation effect on the mediation effect of this path, as seen in Table 10. Meanwhile, the bias-corrected interval under both low government enforcement (−1SD) and high government enforcement (+1SD) does not include 0, so the mediation effect is significant. However, it can be found in the table that the effect value under high government enforcement (0.223) is significantly higher than the effect value under low government enforcement (0.059), as seen in Table 10, so this article concludes that the moderation effect of plastic restriction enforcement on the mediation effect of this path exists.

## Discussion

- Consumers refuse to prioritize the environment over the individual. We assumed that attitudes toward reusable bag consumption share a positive association with intentions, which has been verified by the previous traditional rational paradigm. However, the study shows that both the market environment and individual consumers are constantly changing. When consumers begin to consider their own interests and prioritize their own interests over environmental protection, it is difficult to promote reusable bag consumption behavior despite the positive effects of values, rationality, and attitudes, which is the reason for the gap in attitude toward reusable bag consumption and behavior. It is worth noting that this inconsistency between consumers' words and actions will not be resolved for a long time in the future, because it is considered that consumers have always viewed environmental protection and personal interests as opposites. By default, consumers sacrifice their personal interests to protect the environment, which further explains the fact that the stricter the ban on plastic bags, the more the behavior toward the use of plastic changes.
- Reusable bag consumption behavior is a result of multiple pathways working together. We found that the reasons for reusable bag consumption have a positive effect on consumers' attitudes toward consumption but not on their intentions toward reusable bags. The reason for the abovementioned phenomenon may be the path lock-in effect, which means that consumers will engage in careful and complex considerations when making high-cost decisions. Once consumers find out that the higher cost of reusable bag consumption does not lead to a pleasant consumption experience and that they face use barriers, risk barriers, and value barriers, they will decisively stop their reusable bag consumption behavior. At the same time, according to the consistency theory in psychology (Osgood and Tannenbaum, 1955), consumers who have a reason to engage in reusable bag consumption will try to find reasons to defend their consumption decisions. Thus, a strong reusable bag consumption attitude will further support consumption behavior. In contrast, reasons against consumption do not affect the attitude toward reusable bag consumption but directly and negatively affect consumption behavior. Although BRT assumes that consumers use different psychological processes or paths to make consumption decisions, as consumers who pursue psychological shortcuts enter into a single reason decision mode, the reason against consumption will directly affect the intention of consumption, without the variable of attitude. This also provides a more comprehensive explanation for the inconsistency between consumers' attitudes and behaviors toward substitute plastic behavior.
- We emphasize the guiding function of the value of attitude toward reusable bag consumption. In contrast to the previous research conclusions of scholars, this study found that the consumer values will directly and significantly affect the consumer attitude toward reusable bag consumption (H5). The reasons considered include the following. First, China has been strict about white pollution control and has always been under government regulation and propaganda, which is a unique environment faced by the Chinese consumers. In addition, the production cost has dropped significantly due to the technological progress of degradable plastics, and the price of environmental protection bags is not high. Therefore, consumers do not need to take an in-depth consideration when consuming plastic substitutes. At the same time, the social attributes of the Chinese strong culture may also lead to different results.

## Conclusion

Traditional rational behavior theories fail to enable consumers to agree on consumer attitudes toward reusable bag consumption and behaviors, and BRT bridges the gap between attitude and behavior. In China's unique and strong contextual culture, ABC fully considers the moderation role of contextual variables. Therefore, based on BRT and ABC, we conducted a more extensive research on the Chinese consumers' reusable bag behavior and obtained the following findings: (1) values are the forerunner of behaviors. We emphasize the unique role of reusable bag consumption values. In the consumption process of individual consumers, consumption values play an important role in reusable bag consumption decisions. When established, consumption values can encourage the behavior of reusable bags through rationality or values. (2) The reasons against reusable bag consumption will directly determine whether consumers will buy reusable products. Due to the psychological shortcut of consumers, the reason against consumption will avoid attitude to act directly on consumer behavior. Therefore, exploring the reasons why consumers are reluctant to use reusable bags is an important node step in promoting reusable bag consumption. (3) Plastic generation consumption is a kind of instant consumption. Individual consumers are easily influenced by society and non-formal groups when making consumption decisions. Once a positive reusable bag consumption situation is formed among groups, individual consumers will take the initiative to choose reusable bags to gain group identity.

In view of the above findings, this study puts forward the following suggestions to promote consumers' choice of reusable bag consumption.

First, adherence to effective marketing communications is considered. When promoting their products, each company pays attention to integrating consumers' concerns about reusable bag consumption into their marketing activities, with a two-pronged approach of traditional advertising and new media dissemination: promotion of TV stations' reusable consumption public service advertising and cooperation with the head KOLs of new media platforms to show the daily lives of these bloggers who refuse to plastic bag consumption and pursue reusable bags. Surrounded by traditional media and new media, consumers will continue to strengthen their sense of reusable bag consumption and take reusable bag consumption behavior.

Moreover, enterprises should weaken the reasons against reusable bag consumption. Before the design of reusable products production, enterprises identify on a large scale the demand of consumer preferences, from the reusable product price, use consumer feelings and dig deeper into the consumer' reasons for the current reusable products' not meeting the requirements of consumers and the production of reusable products to meet those requirements, and improve the cost performance of reusable products. It is likely to stimulate the consumer to fulfill the reasons for consumption.

In addition, the government should widely emphasize the implementation of the plastic ban and create a cultural atmosphere conducive to reusable bag consumption. Consumers should be made aware that buying reusable products is detrimental to sustainable development. It is important to create a strong culture of reusable consumption so that consumers are aware that it is a dignified act that can enhance their image and morality in the eyes of others, as well as to have a positive social effect. Simultaneously, to improve reusable product subsidies, retailers' willingness to sell reusable products is increased.

The research limitations of this study provide directions for future research. First, this empirical research only focuses on consumer behavior and reusable bag consumption. Future research can apply BRT and ABC to explore retailers' willingness to accept reusable bag consumption. Second, the design of this research is based on cross-sectional data, which is more prone to social expectation deviation. Future studies can be designed to avoid the appearance of non-essential bias through experimental studies or longitudinal studies. In addition, the role of personality traits, such as innovativeness and diversity seeking, on rationality, attitude, and behavioral intention to consume reusable bags is also a future research direction.

## Data availability statement

The original contributions presented in the study are included in the article/supplementary material, further inquiries can be directed to the correspondingauthor/s.

## Ethics statement

Ethical review and approval was not required for the study on human participants in accordance with the local legislation and institutional requirements. Written informed consent from the patients/participants OR patients/participants legal guardian/next of kin was not required to participate in this study in accordance with the national legislation and the institutional requirements.

## Author contributions

Conceptualization was done by XC. Writing of the original draft and preparation were done by DY. Methodology was done by MX. Writing the review and editing were done by SJ, FL, and QC. All authors have read and agreed to the published version of the manuscript

## Funding

The study was supported by the National Natural Science Foundation of China (Project No. 72164007) and the Postgraduate Research and Practice Innovation Program of Jiangsu Province (KYCX21_1040).

## Conflict of interest

The authors declare that the research was conducted in the absence of any commercial or financial relationships that could be construed as a potential conflict of interest.

## Publisher's note

All claims expressed in this article are solely those of the authors and do not necessarily represent those of their affiliated organizations, or those of the publisher, the editors and the reviewers. Any product that may be evaluated in this article, or claim that may be made by its manufacturer, is not guaranteed or endorsed by the publisher.

## Footnotes

^1^ https://www.mee.gov.cn/ywgz/xcjy/gzcy_27007/202007/t20200714_789277.shtml
